# Comparing the efficacy and safety of two regimens of sequential systemic corticosteroids in the treatment of acute exacerbation of bronchial asthma

**DOI:** 10.4103/0974-2700.66522

**Published:** 2010

**Authors:** Praveen Aggarwal, Sanjeev Bhoi

**Affiliations:** Division of Emergency Medicine, All India Institute of Medical Sciences, New Delhi, India

**Keywords:** Acute asthma, asthma score, hydrocortisone, methylprednisolone, prednisolone

## Abstract

**Background:**

Corticosteroids are commonly used in the management of acute asthma. However, studies comparing various steroids in the management of acute asthma are lacking.

**Objective:**

To compare the efficacy and safety of two treatment regimens – intravenous (IV) methylprednisolone (MP) followed by oral MP and IV hydrocortisone (HC) followed by oral prednisolone in acute bronchial asthma patients.

**Materials and Methods:**

This was a randomized, prospective study performed in the emergency department (ED) of a tertiary care hospital in North India. A total of 94 patients with acute asthma were randomly allocated to either of the two treatment groups: Group A (*n* = 49) or Group B (*n* = 45). Patients in Group A were administered HC 200 mg IV 6-hourly until discharge from the ED, followed by oral prednisolone 0.75 mg/kg daily for 2 weeks. Patients in Group B were administered MP 125 mg IV bolus, followed by 40 mg MP IV 6-hourly until discharge, and then oral MP 0.6 mg/kg daily for 2 weeks. All clinical variables, peak expiratory flow (PEF) and forced expiratory volume in one second (FEV_1_) were assessed at baseline, at 1, 3 and 6 h and at every 6 h thereafter until discharge from the ED. The patients were followed-up after 2 weeks of discharge. The response to treatment was assessed by clinical and spirometric evaluation. Independent *t*-tests and chi-square tests were used to compare the two treatment regimens.

**Results:**

The baseline characteristics were comparable in the two groups. There was a significant improvement in PEF and FEV_1_ within the groups at 2 weeks of treatment when compared to the baseline. At 2 weeks of follow-up, Group B showed significant improvement over Group A in PEF (*P* < 0.0001), FEV_1_ (*P* < 0.0001) and asthma score (*P* = 0.034). There was a significant increase in the blood sugar value at 2 weeks in both the groups. However, the increase was greater in Group A as compared to Group B (*P* < 0.0001).

**Conclusion:**

This study suggests that in acute asthma patients, IV MP followed by oral MP is a more efficacious and safer treatment regimen than IV HC followed by oral prednisolone.

## INTRODUCTION

Acute bronchial asthma is a common presentation to the emergency department (ED) worldwide. In the United States, approximately 1.8 million patients present with acute asthma in the ED every year, of which nearly 500,000 patients are hospitalized.[1] According to the National Family Health Survey-2 (NFHS-2) report, the estimated prevalence of asthma in India is 2,468 per 100,000 persons.[2] Evidence from previous studies suggests that systemic corticosteroid therapy in addition to standard therapy should be administered promptly to patients with features of severe asthma.[3,4]

Most studies conducted on patients with acute exacerbation of bronchial asthma used intravenous (IV) methylprednisolone (MP) initially, followed by oral glucocorticoids (usually MP) for the next 1–8 weeks. The practice patterns in India strongly favor the use of hydrocortisone (HC) followed by oral prednisolone, as this regimen costs lower than the IV MP/oral MP regimen. However, as MP is known to have beneficial pharmacological properties, such as lesser mineralocorticoid potency,[5] greater inhibition of inflammatory cells[6] and better penetration into lung tissue,[7] in comparison to HC and prednisolone, it is highly desirable to study these two regimens in patients with acute asthma. Published literature addressing this scientific question is very limited and conflicting. Although the role of IV corticosteroids in acute asthma is well established, we aim to compare corticosteroids that are most often used in acute asthma in different countries. Therefore, the present study was designed to compare the efficacy and safety of IV MP followed by oral MP with IV HC followed by oral prednisolone in the management of patients with acute exacerbation of asthma.

## MATERIALS AND METHODS

The study was conducted over a period of 6 months (from Jun. 2008 to Nov. 2008) in the ED of a tertiary care hospital in North India. The annual patient attendance in the ED is more than 120,000. Ethical approval was obtained from the Institutional Ethics Committee. The patients’ written informed consent was taken before enrolling them in the study. The consent form was made available in both Hindi and English.

### Study design

The study was a randomized pilot study comparing two regimens of glucocorticoids. The patients were blinded to the initial treatment received in the ED, i.e. IV MP or IV HC. However, they were not blinded to the treatment given at the time of discharge, i.e. oral MP or oral prednisolone. One hundred patients were recruited in this pilot study.

### Eligibility criteria

Patients between 13 and 60 years of age who, during the study period, presented to the ED with moderate [peak expiratory flow (PEF) 50–70% of the predicted value or an asthma score of 8–11] or severe (PEF <50% of the predicted value or an asthma score of 12–15) exacerbation of bronchial asthma were included in the study. Patients below the age of 13 years were managed in the pediatric emergency unit while in patients who were above the age of 60 years, distinguishing between chronic obstructive pulmonary diseases and bronchial asthma might have been difficult at times.

Patients were considered to be asthmatic if they had been previously diagnosed to have bronchial asthma or if the medical history and examination findings were suggestive of bronchial asthma, as defined by the British Thoracic Society (BTS) guidelines and the National Asthma Education and Prevention Program Expert Panel Report 3 guidelines.[8,9] Patients with any of the following conditions were excluded: first episode of wheeze, temperature >38°C, patients with comorbid conditions such as pneumonia, congestive heart failure or interstitial lung disease, use of systemic glucocorticoids within the last 30 days, failure to get consent from patients for participation in the study, requirement of assisted ventilation within one hour of arrival to the ED, failure to use a PEF meter, mild exacerbation (PEF >70% of the predicted value or an asthma score of 5–7) and pregnancy.

### Initial assessment

Patients were assessed within the first 5 min of their arrival to the ED to confirm whether they fulfilled the criteria for inclusion and exclusion. Informed consent was obtained from either the patient or the patient’s relatives. Baseline parameters such as pulse rate (PR), blood pressure (BP), respiratory rate (RR), presence or absence of cyanosis, oxygen saturation, arterial blood gas (ABG) analysis, PEF and forced expiratory volume in one second (FEV_1_) were obtained. PEF and FEV_1_ were recorded using an electronic PEF/FEV_1_ meter (Piko^®^-I; Ferraris Respiratory Europe Ltd., Hertford, UK). Two recordings were taken at each time point (e.g., at baseline, 1 h, 3 h and 6 h, at every 6 h until discharge or admission and then at 2 weeks of follow-up), of which the higher value at each time point was recorded. The ABG analysis was performed using a blood gas analyzer NOVA CCX 1+ (Nova Biomedical Corporation, Waltham, Massachusetts, USA).

Assessment of the “severity of exacerbation” was based on the measurement of PEF and asthma score. This asthma-scoring system [Table 1], a modification of the one published by the National Institutes of Health, rates the severity of an episode according to signs and symptoms.[10] Each patient’s asthma was classified as mild (PEF >70% of the predicted value or an asthma score of 5–7), moderate (PEF 50–70% of the predicted value or an asthma score of 8–11) or severe (PEF <50% of the predicted value or an asthma score of 12–15).

**Table 1:** Method of calculating the asthma score (age >12 years)*

	Asthma scoring		
Variables	1 point	2 points	3 points
Respiratory rate (breaths/minute)	≤23	24–27	≥28
Oxygen saturation (percentage)	>95 with room air	90–95 with room air	<90 with room air or supplemental oxygen
Auscultation	Normal breathing or end-expiratory wheeze	Expiratory wheeze	Inspiratory and expiratory wheeze, diminished breath sounds or both
Retractions	None or intercostal	Intercostal and substernal	Intercostal, substernal and supraclavicular
Dyspnea	Speaks in sentences or coos and babbles	Speaks in partial sentences or utters short cries	Speaks in single words or short phrases or grunts

* THE OVERALL ASTHMA SCORE WAS CALCULATED BY ADDING THE SCORES FOR EACH OF THE FOLLOWING FIVE VARIABLES: RESPIRATORY RATE, OXYGEN SATURATION, AUSCULTATION, RETRACTIONS AND DYSPNEA[10]

### Randomization and medications

After enrolment, the patients were randomly allocated to one of the two treatment groups – Group A or Group B. Block randomization (with blocks of six patients) using the random number table was generated for treatment allocation. Individual random numbers were kept in separate envelopes to maintain allocation concealment.

Group A patients received HC 200 mg IV 6-hourly (Labocort^®^; Laborate Pharmaceuticals Ltd., Panipat, India) till discharge, followed by oral prednisolone 0.75 mg/kg daily (Omnacortil^®^; Macleods Pharmaceuticals Ltd., Mumbai, India) for 2 weeks. Group B patients received MP 125 mg IV bolus (Solumedrol^®^; Pharmacia Italia S.p.A, Italy, marketed by Pfizer Products India Pvt. Ltd., India), followed by 40 mg IV 6-hourly till discharge, and then oral MP 0.6 mg/kg daily (Medrol^®^; Pharmacia Italia S.p.A, marketed by Pfizer Products India Pvt. Ltd.) for 2 weeks.

In addition, each group of patients received standard treatment with nebulization of ipratropium 250 µg and salbutamol 2.5 mg diluted in 3 ml of normal saline initially every 20 min for three doses. Based on the patient’s response to the treatment, subsequent doses of bronchodilators were administered. Except for IV corticosteroids, all other medications were administered by the ED physicians who were not part of the study. Antibiotics were given if deemed necessary. Along with medications, all patients were administered oxygen at 4–6 L/min by nasal cannulae or face masks. Ventilator support was instituted at the discretion of the ED physicians as per the patient’s condition.

### Assessment of response

All clinical variables, PEF and FEV_1_, were assessed at baseline and during the course of stay in the ED at 1, 3 and 6 h and every 6 h thereafter until discharge or admission. The ABG analysis was performed at baseline and was repeated if deemed necessary by the attending ED physicians. The decision to discharge a patient was taken by the attending emergency physician. It was usually made once the PEF was >65% of the predicted value.

The patients were followed-up after 2 weeks for response to treatment (by clinical and spirometric evaluation) and any side-effects. A revisit to any hospital due to recurrence of acute exacerbation during the 2 weeks of follow-up was also recorded.

Primary and secondary endpoints were ascertained to compare the efficacy of the two treatment regimens. The primary endpoint was “treatment failure,” which was considered if any of the following criteria was met: need for mechanical ventilation, need for hospitalization, mortality within 2 weeks of initial presentation, readmission for acute exacerbation within 2 weeks of discharge or physician-directed intensification of pharmacologic therapy between discharge from the hospital and follow-up at 2 weeks.

Secondary endpoints were change in FEV_1_ and PEF during the patient’s stay in the ED and at 2 weeks of follow-up, change in asthma score at 2 weeks of follow-up, length of stay in the ED (if discharged from the ED) and length of stay in the hospital (if admitted to the inpatient care). In addition, the nature and severity of the adverse effects occurring within 2 weeks were observed and compared in the two treatment groups.

### Statistical methods

Because a literature search identified just one study comparing the efficacy between MP and HC,[11] the current study was conducted as a pilot study to help estimate the sample size for a confirmatory study. Hence, a purposive sampling methodology was used and a sample size of 100 patients was selected for the study.

Descriptive statistics (mean, standard deviation and median) were calculated for each quantitative variable. Independent *t*-test was utilized for comparing the means between the groups. An appropriate non-parametric test such as the Mann-Whitney *U*-test was utilized whenever normality assumption was not satisfied. Between-group comparisons of categorical variables were performed using chi-square (χ^2^) tests. Within-group comparisons were performed using paired *t*-test to test the difference between baseline and 2 weeks. Repeated measures analysis of variance (ANOVA) was used to test the difference between the means at different time points within the group. Tukey’s studentized range (*q*) test was used for multiple comparisons of FEV_1_ and PEF between different time points. The results were considered significant at the 5% level (*P* < 0.05).

The data were entered into a database program (Microsoft Excel, version 2000, Microsoft, Redmond, WA, USA) and were imported into a statistical software package (SAS version 9.1.3 Service pack 4, SAS institute Inc., Cary, NC, USA) for analysis.

## RESULTS

**Figure 1:**
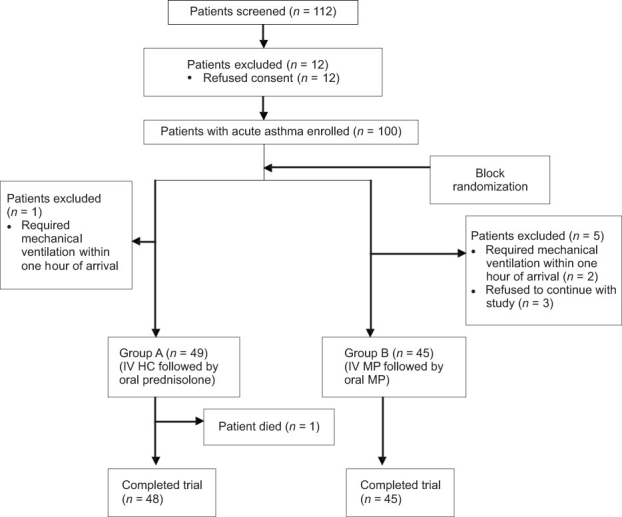
Flow diagram showing enrolment and allocation of patients to the two study groups

One hundred and twelve patients were screened, of which 12 refused to give consent. A total of 100 patients were recruited and randomly allocated to either of the two treatment groups [Group A (*n* = 49) and Group B (*n* = 45)] [Figure 1]. Six patients were excluded from the study within 1 h of randomization due to various reasons. Therefore, only 94 patients were finally subjected to efficacy and safety analysis.

At baseline, the patients in the two groups did not differ significantly with regard to age, history of smoking, duration of asthma, medications used for chronic phase, duration of present episode of acute asthma and medications received before presentation to the ED [Table 2]. Also, there was no significant difference in the ABG values between the groups at baseline. None of the patients had used oral or parenteral steroids in the last 30 days. The clinical features of acute exacerbation in these patients mainly included increased cough with expectoration, dyspnea, intercostal/substernal/suprasternal retractions and presence of rhonchi.

**Table 2:** Baseline characteristics of the patients in the two study groups*

	Group A (*n* = 49)	Group B (*n* = 45)	*P*-value
Age (years)†	45.0 (14.76)	45.2 (14.92)	0.954
Men:women‡	31:18	25:20	0.447
Duration of asthma (years)†	12.0 (6.64)	13.7 (9.49)	0.331
Chronic use of medications for control of asthma‡	42 (49)	39 (45)	0.893
Smokers‡	4 (8.2%)	4 (8.9%)	1
Any other comorbid illness‡	5 (10.2%)	6 (13.3%)	0.881
Duration of present attack (days)†	1.9 (0.65)	1.9 (0.91)	0.645
Medications received for acute exacerbation before ED visit‡	49 (49)	43 (45)	0.136
Oxygen saturation (%)	90.0 (2.18)	89.5 (2.12)	0.298

* GROUP A: IV HC FOLLOWED BY ORAL PREDNISOLONE; GROUP B: IV MP FOLLOWED BY ORAL MP;

† MEAN (SD);

‡ NO. OF PATIENTS; TEST DONE AT 5% SIGNIFICANCE LEVEL AND *P* < 0.05 INDICATES SIGNIFICANCE

Pulse rate, BP (diastolic and systolic), RR, PEF, FEV_1_ and asthma score at baseline were not significantly different between the groups.

After treatment, PR, RR, PEF, FEV_1_ and asthma score showed significant improvement in both the groups at the time of discharge as well as at 2 weeks of follow-up [Table 3].

**Table 3:** Difference between groups at baseline and at the end of 2 weeks [PEF, FEV_1_, asthma score, pulse rate, blood pressure (systolic and diastolic), respiratory rate, blood sugar]

	Group	Baseline mean (SD)	2 weeks mean (SD)	Change from baseline mean (SD)
PEF (L/min)	Group A (*n* = 49)	70.7 (20.38)	222.8 (45.23)	152.1 (45.74)
	Group B (*n* = 45)	67.9 (19.77)	257.6 (33.68)	189.7 (34.42)
	*P*-value	0.503		<0.0001
FEV_1_ (L)	Group A (*n* = 49)	0.5 (0.16)	2.0 (0.35)	1.4 (0.37)
	Group B (*n* = 45)	0.5 (0.17)	2.3 (0.33)	1.8 (0.31)
	*P*-value	0.422		<0.0001
Asthma score	Group A (*n* = 49)	12.1 (1.05)	5.1 (0.87)	-6.9 (1.28)
	Group B (*n* = 45)	12.4 (0.71)	5.0 (0)	-7.4 (0.71)
	*P*-value	0.113		0.034
PR (beats/min)	Group A (*n* = 49)	105.3 (2.87)	85.8 (4.88)	-19.6 (5.3)
	Group B (*n* = 45)	106.5 (2.76)	83.7 (3.51)	-22.8 (4.59)
	*P*-value	0.053		0.003
BP diastolic (mmHg)	Group A (*n* = 49)	78.7 (7.14)	83.5 (6.22)	4.8 (7.54)
	Group B (*n* = 45)	81.6 (10.35)	80.2 (4.9)	-1.4 (9.7)
	*P*-value	0.115		0.0009
BP systolic (mmHg)	Group A (*n* = 49)	125.8 (14.27)	129.1 (10.27)	2.8 (12.41)
	Group B (*n* = 45)	128.3 (18.98)	123.6 (9.2)	-4.7(14.12)
	*P*-value	0.468		0.0087
RR (beats/min)	Group A (*n* = 49)	30.1 (3.03)	16.7 (2.23)	-13.4 (2.6)
	Group B (*n* = 45)	31.1 (3.81)	16.3 (1.44)	-14.9 (3.22)
	*P*-value	0.14		0.015
Blood sugar (mg/dl)	Group A (*n* = 49)	98.2 (12.06)	135.0 (28.01)	36.7(32.17)
	Group B (*n* = 45)	113.6 (20.51)	122.2 (12.55)	8.6 (25.89)
	*P*-value	<0.0001		<0.0001

PEF: PEAK EXPIRATORY FLOW; FEV_1_: FORCED EXPIRATORY VOLUME IN ONE SECOND, BP: BLOOD PRESSURE; PR: PULSE RATE, RR: RESPIRATORY RATE; TEST DONE AT 5% SIGNIFICANCE LEVEL AND *P* <0.05 INDICATES SIGNIFICANCE

**Figure 2:**
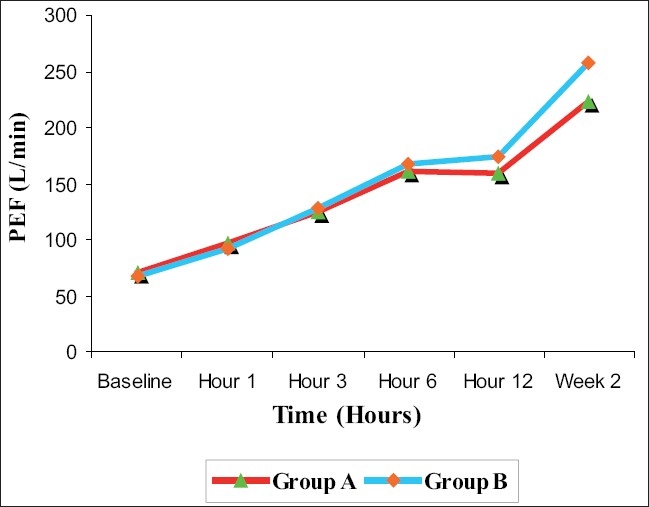
Mean peak expiratory flow (l/min) at various time points by treatment groups in acute asthma

**Figure 3:**
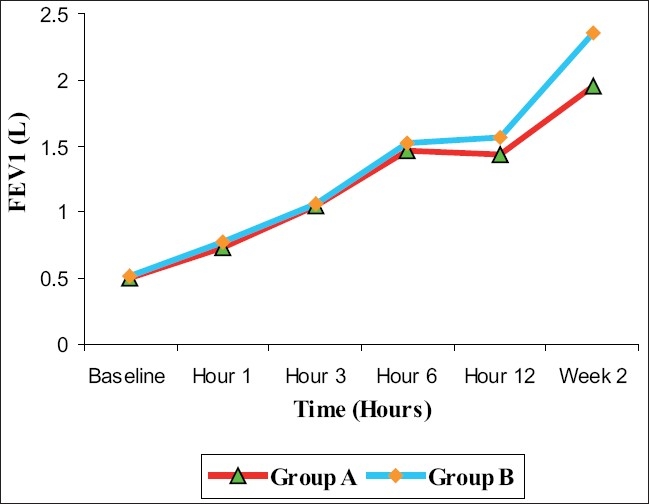
Mean forced expiratory volume in one second at various time points by treatment groups in acute asthma

There was a steady and significant increase in the mean PEF values within each study group from baseline to 6 h of stay in the ED, with the highest reading at 6 h (*P* < 0.0001). Group A patients had a mean PEF of 160.0 (± 40.44) L/min at 6 h while Group B patients had a mean PEF of 167.6 (± 33.39) L/min at 6 h. When the mean PEF values of the two groups were compared, there was no significant difference in their change at various time points in the ED (*P* = 0.431 at 1 h, *P* = 0.704 at 3 h, *P* = 0.341 at 6 h) [Figure 2]. Similar results were observed in the mean FEV_1_ also (*P* = 0.41 at 1 h, *P* = 0.874 at 3 h, *P* = 0.609 at 6 h) [Figure 3].

Increase in PEF and FEV_1_ were not statistically different between the two groups at the time of discharge. However, a significant difference in PEF and FEV_1_ was observed between the two groups at 2 weeks, with Group B showing greater improvement in PEF and FEV_1_ than Group A (*P* < 0.001; Table 3).

Mean duration of stay in the ED was 10.2 (± 6.49) h in Group A and 8.9 (± 5.99) h in Group B, with no significant difference between the groups (*P* = 0.32). One patient in Group A required hospital admission. There was no significant difference between the groups in the requirement of additional doses of nebulization during the first 6 h of acute exacerbation (*P* = 0.136). Also, there was no significant difference between the two groups (*P* = 0.74) with regard to additional medications (oral bronchodilators and antibiotics) given by the attending emergency physicians at the time of discharge.

At baseline, the mean blood sugar values were 98.2 (± 12.06) mg/dl and 113.6 (± 20.51) mg/dl in Group A and Group B, respectively (*P* < 0.0001). Following treatment for 2 weeks, the mean blood sugar values were significantly greater in Group A [135.0 (± 28.01) mg/dl] as compared to Group B [122.2 (± 12.55) mg/dl] (*P* < 0.0001). Within the groups, there was a significant difference in blood sugar values at 2 weeks from baseline (Group A: *P* < 0.0001; Group B: *P* = 0.031). Diastolic BP increased significantly from 78.7 (± 7.14) mmHg to 83.5 (± 6.22) mmHg (*P* < 0.0001) in Group A while systolic BP decreased from 128.3 (± 18.98) mmHg to 123.6 (± 9.2) mmHg (*P* = 0.032) in Group B. The ABG analysis was performed only at baseline, as based on the patient’s clinical condition; a repeat ABG was not required in most patients.

In Group A, two patients (4.17%) had relapsed within 2 weeks. One patient relapsed on the 14^th^ day of follow-up; his asthma score was 11 at the time. Another patient relapsed on the 10^th^ day and was treated in another hospital. He recovered in 6 h, after which he continued with the tablets prescribed in the study. Another patient (2.04%) in Group A required ventilator support due to respiratory muscle fatigue; he died 48 h after arrival to the ED.

At 2 weeks of follow-up, the following adverse events were observed in patients in the two groups: (a) abdominal distension and constipation each in two patients (8.33%) in Group A, (b) burning pain in the epigastrium in two patients in Group A (8.33%) and in one patient in Group B (2.22%) and (c) facial puffiness in seven patients (14.58%) in Group A and in three patients (6.67%) in Group B.

## DISCUSSION

It is important to achieve rapid improvement in lung function during the first few hours of severe acute asthma as it is most affected during this time. A prompt corticosteroid therapy is therefore desirable at this stage,[12,13] although its action may be delayed by at least 4 h.[14] International guidelines such as the Global Initiative for Asthma (GINA)[14] and the BTS guidelines[8] recommend early use of systemic corticosteroids in acute asthma as it has been shown to reduce the hospital admission rate, length of hospitalization and relapse rate after discharge. A short course of oral corticosteroids is recommended in acute bronchial asthma patients after discharge from the ED as it reduces the relapse rate and the need for using reliever inhalers with no major adverse effects.[3,15]

There are inconsistencies in the various reported studies regarding the role of different corticosteroids in bronchial asthma as the efficacies of corticosteroids may vary due to their dissimilar antiinflammatory potencies.[16] While MP may have a greater antiinflammatory potency, longer duration of action and lesser sodium-retaining properties as compared to prednisolone and hydrocortisone,[11] there are very few studies in the published literature comparing different corticosteroid regimens in acute exacerbation of asthma.

Gordon *et al*. (1984) evaluated the effects of two doses of MP of 125 mg or 60 mg against HC of 100 mg on serum immunoglobulin E levels (IgE). IgE levels decreased significantly in patients treated with the higher dose of MP (125 mg), while there was not much of a decrease in the IgE levels in patients treated with either HC or with a lower dose of MP (60 mg).[17] In another study, 14 patients with acute bronchial asthma were treated with HC (100 mg IV q 6-hourly), MP (20 mg IV q 6-hourly) or dexamethasone (3.75 mg IV q 6-hourly). No difference was found in the efficacies of any of these three drugs up to 72 h.[18] In a study by Hall *et al*. (1996), HC was found to be more effective than MP in reducing stay in the asthma unit in patients with acute severe asthma.[11] However, HC was administered at the dose of 200 mg IV 4-hourly, while MP was given at the dose of 125 mg IV 12-hourly. None of these studies have compared the effects of sequential regimen of intravenous steroids in ED followed by a short course of oral steroids, which is the recommended practice.

The present study aimed to compare the safety and efficacy of two treatment regimens – IV HC followed by oral prednisolone and IV MP followed by oral MP in acute asthma patients. The study showed that there was a significant improvement in the lung functions within few hours of administration of IV corticosteroids in the ED. However, there was no significant difference in the improvement observed during the first 6 h between the two groups. At 2 weeks of follow-up, the improvement in lung function with oral MP was significantly greater than with oral prednisolone. This difference at the end of 2 weeks could be explained by multiple properties of MP. Methylprednisolone is known to have a greater antiinflammatory potency, with lesser mineralocorticoid action as compared to prednisolone.[11] Specifically, it has been shown to have much greater effects on specific cells involved in inflammation (e.g., peripheral blood mononuclear cells) as compared to prednisolone.[6] Similarly, some animal studies have shown that MP attains much higher concentrations in lung tissue as compared to prednisolone over a period of time after a single dose. This may lead to increase in the availability of the drug at its site of action.[7] Patients in Group B had a slightly shorter duration of stay in the ED as compared to those in Group A, although the difference was not significant.

In the present study, blood glucose values increased in both the groups at 2 weeks of follow-up; however, this increase was more significant in Group A as compared to Group B (*P* < 0.0001 vs. *P* = 0.031). This increase could be attributed to administration of oral prednisolone for 2 weeks in Group A. This finding supports the findings from various other studies that have shown the hyperglycemic effects of prednisolone.[19] Adverse events data showed that IV MP/oral MP may be comparatively safer than IV HC/oral prednisolone, although this can be confirmed only with the inclusion of a larger sample size in further studies. In Group A, three patients reached the primary endpoint, of which two patients had relapsed within 2 weeks, and one died.

There are some limitations to the present study. The sample size of the study was small; however, as mentioned, it was a pilot study. The second limitation is that this study was not double-blinded and there could have been some bias by the physicians involved in conducting the study. The study was performed in the emergency unit with an average staff of 10 physicians per emergency shift with rotation of shifts on a daily basis. Except for the steroid use, other treatments were decided by the emergency physicians who were not part of the study. They were also responsible for deciding about admission or discharge of patients and, therefore, individual physician’s bias is unlikely to have affected the study results. The third limitation is the doses of MP and HC/prednisolone used in the study. Bowler *et al*. (1992) showed, in their study, that a lower dose of IV HC (50 mg) four times a day for 2 days followed by a low-dose oral prednisone is as effective in resolving acute severe asthma as are 200 mg or 500 mg doses of HC, followed by higher doses of prednisone.[20] The BTS guidelines[8] also recommend a lower dose of HC (100 mg IV 6-hourly) followed by 40–50 mg of prednisolone for 5 days or until recovery of acute asthma. The HC and prednisolone doses used in the study were based on the recommended doses by Taylor (2003).[21] Methylprednisolone was used in doses equivalent to HC doses. Based on the BTS guidelines, lower doses for HC/MP and shorter duration of oral MP/prednisolone could have been considered in this study as, apart from the problem of high cost, high doses of corticosteroids may cause more steroid-related side-effects than low doses. Another limitation of the study was the exclusion of patients below 13 years of age, although pediatric patients constitute a major group who present with acute exacerbation of asthma. Therefore, the sample population considered for the study might not be an appropriate sample to effectively represent the true population of acute asthma patients.

To conclude, the present study has shown a higher efficacy and safety of IV MP followed by oral MP as compared to IV HC followed by oral prednisolone in acute asthma patients. Based on the results of this pilot study, IV MP followed by oral MP, despite its higher cost, may form the treatment of choice among the two regimens for acute bronchial asthma. Nevertheless, the plasma and biological half-life of MP is two–three times more than HC,[22] so less-frequent dosing schedule could be considered for MP to conceivably reduce the total cost of treatment.
